# Mr. Earnest, on Typhus Fever

**Published:** 1806-03

**Authors:** Robert Earnest

**Affiliations:** House Surgeon to the Sheffield General Infirmary


### *To the Editors of the Medical and Physical Journal.*

Gentlemen,

It is a matter of the greatest importance, where it is practicable, to ascertain (after the death of a patient who had been labouring under any disease of an obscure nature) the state of the different viscera, and more particularly such parts in which were seated, the pains, or other grievances that the patient complained of during the progress of affliction. By such anatomical investigations in search of what had been destructive to life, it is highly probable, new light may be thrown upon the treatment of many intricate maladies, which too often confound, and as it were, bid defiance to the abilities and skill of the most learned and eminent practitioners. I am sorry to say that the annexed individual case, wherein so much disease existed, that had there been a pretty accurate knowledge of the state of things, I very much doubt that it would have been in the power of art to have accomplished a cure: and although the following history of this extraordinary case does not point out, or allude to any success of the remedies employed therein, but on the contrary, the case itself will appear to have been irremediable; yet the publishing of such cases may, very possibly, assist in pointing out some new and valuable documents for future practice, when diseases of a similar nature again occur; and most certainly the improvement of the profession must necessarily be deduced from such communications coming before the public, and from which, of course, new discoveries in the healing art are to be expected.

I am, &c.

ROBERT EARNEST,

*Feb.* 10, 1806. House Surgeon to the Sheffield General Infirmary.

---

### CASE.

D. D. a youth, nineteen years of age, with a weakly constitution, low in stature, and his complexion florid and delicate. In the year 1795, he was attacked with typhus fever, caught by contagion.

The fever continued for a considerable length of time, which emaciated the patient very materially, and left him in a state of extreme debility, which he never recovered. He was ailing several years, during which time a numerous train of anomalous symptoms came on, which rendered him exceedingly nervous and irritable; so much so, that the least noise imaginable would alarm and agitate him very distressingly for a short time, and which always brought on, perhaps, the worst palpitation of the heart that ever was either heard or felt; the slightest motion or exertion sometimes would also bring on this truly painful symptom; and his fear of molestation was so great, that his mind was scarcely ever at ease, unless the door of his apartment was bolted.

It would be a difficult task to detail a vast number of the more trifling symptoms that this patient used to complain of, but I will here endeavour to enumerate the more prominent ones, and such as most required the attention of the medical attendant; namely, the face was highly flushed with a deep red colour; the pulse, morning and evening, was generally at 82 or 83; but upon the least motion of the body it would become rapid, very irregular, and would sometimes intermit; the fauces and sides of the tongue were inflamed and excoriated, and the tonsils were nearly destroyed; destroyed; the appetite was not much impaired, but the bowels were always costive, accompanied with constant heat and pain of the anus and rectum; there was often a very painful head-ach, and, to use the patient's own words, 'with a sense of coldness in the brain;' the respiration was laborious, short, and quick; the speech and voice were very much affected; and whenever he endeavoured to talk, he was obliged to pause a little between every two or three words; a short and troublesome cough, without expectoration; and, as it was before observed, extreme debility in motion. There was great watchfulness; and when he did sleep, he sometimes awoke in a very deplorable state, as if he was dying; and after he had recovered from such an attack, he would describe his feelings at the time in the following manner. "I felt great oppression about my heart, and had not the power to breathe, or to move in the least possible degree; my senses failed me for a few moments, and it is with the greatest difficulty that I catch my breath again, and that at these times I am fully persuaded that life for a short while is suspended."

He could not lie in a horizontal posture, consequently he was obliged to be placed in bed in a half sitting position, reclining a little backwards.

With these symptoms the patient suffered much, and lingered for upwards of five years; and in the end, when he had arrived at the age of 24, his complaints terminated in general anasarca and death.

The patient during his very long and painful illness, had been attended at different times by several different physicians and surgeons; but from the very obscure nature of his complaints, none of them were able to afford him any lasting relief; nor will their want of success appear at all surprising, when the reader is made acquainted with the morbid changes and appearances that had taken place in the structure of some of the viscera contained in the thorax and abdomen; of which the following account of the subsequent dissection will sufficiently testify.

1. The contents of the thorax were examined, and here the right lobe of the lungs adhered firmly to the pleura in several places, and this side of the chest was nearly filled with water: on the left side, the lung appeared to be compressed into a much smaller compass than natural, by the heart, which had become considerably enlarged; I may say, to nearly twice its natural dimensions, and the apex of it, as it were, was tilted up and pressed against the the side. The pericardium adhered closely to the heart universally. On examining the blood vessels going immediately from the heart, there did not appear any thing like aneurism; but they appeared larger and more dilated than is generally seen in a healthy subject. The pulmonary artery in that part just before it sends off branches to the lungs, &c. shewed signs of approaching ossification, as also did the two bronchials.

2. In the abdomen, the liver was somewhat enlarged, and was darker in colour than is natural; but upon cutting into its substance there did not appear any apparent disease, neither were there any adhesions of it to the peritoneum, or any other part. The stomach and bowels were fully distended with air, and appeared to be in a state of inflammation, and the blood-vessels upon their surface were beautifully injected with blood of the brightest scarlet colour, as also were those of the omentum.

The coats of the rectum were much thickned, but more particularly so towards the extremity of it, where there was evident beginning schirrosity, and the passage at the anus was considerably contracted.*

All the other parts were natural.

---

### On the Means of supplying the Poor more effectually with Medical Assistance in the Country Parts of Ireland.

It is now several years since the Legislature of this country, taking into consideration the distressed and helpless

---

* The bowels in this case being always very costive, the patient could never part with stools without the aid of domestic glysters, once and twice a day; as, sometimes, the enema that was injected in the morning did not come away during the day, it was therefore necessary to repeat another in the evening. The frequent introduction of the glyster-pipe, although a small one, never failed to produce a great deal of pain for two or three hours together; and the patient was so fearful of this operation, that so long as he was able himself to perform it, he never would suffer any one else to do it for him; accordingly he had an apparatus made for that purpose, after the manner of a French glyster syringe, which he could use in a sitting posture very conveniently.

From the almost constant pain and soreness he felt in the rectum and parts adjacent, he was always of opinion that these parts would be found in a diseased state; and it clearly appears from the above statement, that his ideas, in this particular, were verified.

